# APRICOT-Mamba: Acuity Prediction in Intensive Care Unit (ICU): Development and Validation of a Stability, Transitions, and Life-Sustaining Therapies Prediction Model

**DOI:** 10.21203/rs.3.rs-4790824/v1

**Published:** 2024-08-06

**Authors:** Miguel Contreras, Brandon Silva, Benjamin Shickel, Andrea Davidson, Tezcan Ozrazgat-Baslanti, Yuanfang Ren, Ziyuan Guan, Jeremy Balch, Jiaqing Zhang, Sabyasachi Bandyopadhyay, Tyler Loftus, Kia Khezeli, Subhash Nerella, Azra Bihorac, Parisa Rashidi

**Affiliations:** 1 Department of Biomedical Engineering, University of Florida, Gainesville, FL, USA; 2 Department of Electrical and Computer Engineering, University of Florida, Gainesville, FL, USA; 3 Division of Nephrology, Department of Medicine, University of Florida, Gainesville, FL, USA; 4 Department of Surgery, University of Florida, Gainesville, FL, USA; 5 Department of Medicine, Stanford University, Stanford, CA, USA; 6 Intelligent Clinical Care Center (IC3), University of Florida, Gainesville, FL, USA

**Keywords:** state space, acuity, transformers, mortality, intensive care unit

## Abstract

On average, more than 5 million patients are admitted to intensive care units (ICUs) in the US, with mortality rates ranging from 10 to 29%. The acuity state of patients in the ICU can quickly change from stable to unstable, sometimes leading to life-threatening conditions. Early detection of deteriorating conditions can assist in more timely interventions and improved survival rates. While Artificial Intelligence (AI)-based models show potential for assessing acuity in a more granular and automated manner, they typically use mortality as a proxy of acuity in the ICU. Furthermore, these methods do not determine the acuity state of a patient (*i.e.*, stable or unstable), the transition between acuity states, or the need for life-sustaining therapies. In this study, we propose APRICOT-M (Acuity Prediction in Intensive Care Unit-Mamba), a 1M-parameter state space-based neural network to predict acuity state, transitions, and the need for life-sustaining therapies in real-time among ICU patients. The model integrates ICU data in the preceding four hours (including vital signs, laboratory results, assessment scores, and medications) and patient characteristics (age, sex, race, and comorbidities) to predict the acuity outcomes in the next four hours. Our state space-based model can process sparse and irregularly sampled data without manual imputation, thus reducing the noise in input data and increasing inference speed. The model was trained on data from 107,473 patients (142,062 ICU admissions) from 55 hospitals between 2014–2017 and validated externally on data from 74,901 patients (101,356 ICU admissions) from 143 hospitals. Additionally, it was validated temporally on data from 12,927 patients (15,940 ICU admissions) from one hospital in 2018–2019 and prospectively on data from 215 patients (369 ICU admissions) from one hospital in 2021–2023. Three datasets were used for training and evaluation: the University of Florida Health (UFH) dataset, the electronic ICU Collaborative Research Database (eICU), and the Medical Information Mart for Intensive Care (MIMIC)-IV dataset. APRICOT-M significantly outperforms the baseline acuity assessment, Sequential Organ Failure Assessment (SOFA), for mortality prediction in both external (AUROC 0.95 CI: 0.94–0.95 compared to 0.78 CI: 0.78–0.79) and prospective (AUROC 0.99 CI: 0.97–1.00 compared to 0.80 CI: 0.65–0.92) cohorts, as well as for instability prediction (external AUROC 0.75 CI: 0.74–0.75 compared to 0.51 CI: 0.51–0.51, and prospective AUROC 0.69 CI: 0.64–0.74 compared to 0.53 CI: 0.50–0.57). This tool has the potential to help clinicians make timely interventions by predicting the transition between acuity states and decision-making on life-sustaining within the next four hours in the ICU.

## 1. Introduction

On average, more than 5 million patients are admitted annually to intensive care units (ICUs) in the US [1], with mortality rates ranging from 10 to 29% depending on the patient’s age, comorbidities, and illness severity [2]. Patient acuity implies the categorization of patients based on their care needs [3]. During an ICU stay, the acuity state of patients can quickly change, leading to life-threatening conditions. Early detection of such deteriorating conditions can assist in more timely interventions and improved survival rates.

Given the frequent pace of monitoring in the ICU, many physiological and clinical measurements are recorded as time series, such as the vital signs, laboratory test results, medications, and assessment scores. The rich temporal data, in conjunction with static patient characteristics (*e.g.*, age, gender, or comorbidities), provide opportunities for implementing real-time approaches for assessing patient acuity and augmenting clinicians’ decisions. Several scores are currently used to assess patient acuity among ICU patients, such as Sequential Organ Failure Assessment (SOFA) [4], Acute Physiology And Chronic Health Evaluation (APACHE) [5], Simplified Acute Physiology Score (SAPS) [6], [7], and Modified Early Warning Score (MEWS) [8]. However, these scores have several limitations including manual calculation and only providing a score at one point in time. More recent approaches have explored the potential use of neural network models to employ the vast amount of Electronic Health Record (EHR) data for automated acuity assessment. Many approaches have focused on mortality prediction as a proxy for real-time patient acuity scores [9], [10], [11] and have been shown to outperform manual scores while providing more timely and frequent acuity assessments. Despite their high performance and granularity, many existing artificial intelligence (AI) methods have the limitation of solely predicting mortality risk. Developing scores that capture a broader spectrum of patient acuity states beyond mortality risk can provide a more complete picture and opportunities for timely interventions.

A recent computational phenotyping method developed by Ren et al. [12] annotated patient acuity states based on the use of four life-sustaining therapies: mechanical ventilation (MV), massive blood transfusion (BT) (defined as at least 10 units in the past 24 hours), vasopressor (VP) administration, and continuous renal replacement therapy (CRRT). A significant number of patients in the ICU require MV, with an incidence rate of 20–40% [1], while 30–50% of ICU patients require BT [13]. Vasopressors (VP) are used to manage blood pressure in about 27% of ICU patients [14], and CRRT is needed in patients with chronic kidney disease or acute kidney injury, up to 14% of ICU patients [15]. Ren et al. considered patients unstable if they required at least one of the four life-supportive therapies (MV, BT, VP, CRRT); if not, they were considered stable. The stability state predictions, together with the discharge and mortality predictions, provide a more complete picture of patient acuity.

Recent advances in AI have shown that the Transformer architecture [16] can be very effective for time-series classification tasks [17], [18]. Specifically, this architecture has been adapted for clinical time series with the development of novel embeddings that can handle the irregular sampling of this type of data [10], [19]. The emergence of state space models (SSM) has recently provided an alternative to the Transformer architecture with hardware efficient design, increased speed and performance on tasks with longer sequence lengths while using fewer parameters [20]. We hypothesize that using SSM models could potentially lead to improved performance in patient acuity prediction compared to the existing deep learning methods.

In this study, we propose APRICOT-M (Acuity Prediction in Intensive Care Unit-Mamba), a novel SSM model to predict real-time acuity, defined as stable, unstable, deceased, or discharged, in the ICU. APRICOT-M uses temporal data from the preceding four hours in the ICU along with static patient characteristic data obtained at admission time to predict patient acuity within the next four hours in the ICU. Specifically, the algorithm predicts the probability of a patient being stable or unstable within the next four hours, along with the probability of mortality or discharge. Furthermore, it predicts transitions between acuity states (*i.e.*, the transition from stable to unstable and vice versa) and the need for three life-sustaining therapies (MV, VP, and CRRT) within the next four hours. Our model is engineered to not require data imputation for missing values in temporal data. This design choice helps in reducing input noise and optimizing the model for real-time deployment. The APRICOT-M model was developed using data obtained from patients who received care at the University of Florida Health (UFH) Shands Hospital between 2014–2017 in conjunction with data from 54 hospitals from the electronic ICU Collaborative Research Database (eICU) [21]. The model was then externally validated on data from hospitals not used in the development phase consisting of the remaining 143 hospitals from eICU and data from the Medical Information Mart for Intensive Care (MIMIC)-IV [22], and temporally validated on data from a period not used in the development phase consisting of UFH data between 2018–2019. Finally, prospective validation was performed with data acquired prospectively from UFH [23] between May 2021 and October 2023. Additionally, a Transformer variant of the model, APRICOT-T, was also developed and used along with three baseline models to demonstrate the advantages of the novel SSM architecture. To our knowledge, the proposed APRICOT-M model is the first real-time acuity prediction tool for patients in the ICU that incorporates the prediction of stable and unstable states, mortality, and discharge, transition in patient acuity states, and the need for life-sustaining therapies, while being extensively validated on three large datasets externally, temporally, and prospectively. Furthermore, it is the first SSM model application to clinical data.

## 2. Methods

### 2.1 Data and Study Design

**Fig. 1 |:**
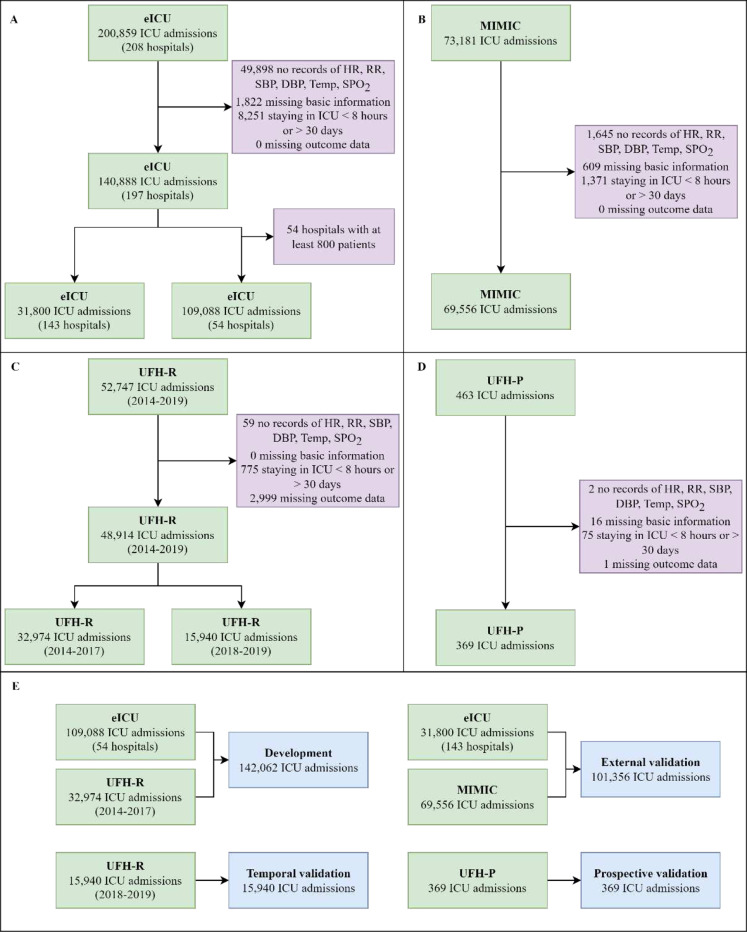
Cohort flow diagram. The (A) eICU, (B) MIMIC, (C) UFH-R, and (D) UFH-P datasets. (E) Final datasets were assembled from the four datasets for development (training and validation), external validation, temporal validation, and prospective validation. We created the development dataset by pooling data from 52 random eICU hospitals with data from UFH-R between 2014–2017. Data from the remaining 146 hospitals at eICU were pooled with data from MIMIC to form the external validation set. The temporal validation dataset was created from UFH-R between 2018 and 2019. The prospective validation dataset was created from real-time data in the UFH-P dataset.

Three databases were used in this study: UFH, MIMIC, and eICU (cohort diagrams in Fig. 1). The UFH dataset was collected in both a retrospective (UFH-R) and prospective (UFH-P) manner, while the MIMIC and eICU datasets were retrospective. The UFH-R dataset includes adult patients admitted to the ICUs at the University of Florida (UF) Health Shands Hospital, Gainesville, between 2014 and 2019, while UFH-P dataset was obtained from adult patients admitted to the ICU at UFH between May 2021 and October 2023 (UFH-P) using our real-time platform. Specifically, UFH-P data was collected and processed on a daily basis. Then, all data from the specified time period was compiled to construct the dataset. The MIMIC dataset is a publicly available dataset collected at the Beth Israel Deaconess Medical Center from 2008 to 2019 [22]. The eICU dataset contains data from ICU patients from 208 hospitals in the Midwest, Northeast, Southern, and Western regions of the US from 2014 to 2015 [21]. In all four datasets, patients were excluded if they had missing records of basic information (*i.e.*, age, discharge location, sex, race, or BMI), outcomes (*i.e.*, acuity states), or missing at least one of six routine vital signs: heart rate (HR), respiratory rate (RR), systolic blood pressure (SBP), diastolic blood pressure (DBP), body temperature (Temp), and oxygen saturation (SPO_2_). Also, patients who stayed in the ICU for less than 8 hours or more than 30 days were excluded. The lower bound was chosen to allow for enough data for one prediction and outcome validation (4-hour observation and subsequent 4-hour prediction window), while the upper bound was chosen to exclude long admissions that could bias model training due to increased number of 4-hour intervals pertaining to single patients.

Data from UFH-R between 2014 and 2017 and data from 54 hospitals from the eICU database were used to create the development set. This set was used to train and tune the APRICOT-M model. Data from MIMIC and the remaining 143 hospitals in eICU were used for external validation to evaluate the generalizability of APRICOT-M to diverse hospital settings. Hospitals from eICU used for development were selected if their cohort contained at least 800 patients to have the largest number of patients possible in the fewest number of hospitals. This setup allowed for a larger number of hospitals to be used in the external validation set. Data from UFH-R between 2018 and 2019 was used for temporal validation to evaluate generalizability to different years of admission. Data from UFH-P was used for prospective validation to evaluate the application of APRICOT-M to a real-time setting.

The study design consisted of six steps: development, calibration, validation (*i.e.*, external and temporal), subgroup analysis, interpretation, and prospective evaluation. This study follows the Transparent Reporting of a multivariable prediction model for Individual Prognosis Or Diagnosis (TRIPOD) Statement [24] (checklist available in Supplementary Material). First, we developed and tuned the APRICOT-M model on the development dataset (80% training, 20% validation), followed by calibration using a random sampling of 10% of each validation dataset to obtain a more accurate risk probability of each outcome. Next, the model was externally validated on hospitals not included in the development stage and temporally validated on data from years that were not included in the development set. We also performed a subgroup analysis of model performance based on age group (18–60 vs over 60), sex, and race, accompanied by detailed interpretability analyses, to find the most relevant predictors correlated with increased patient acuity. Finally, prospective model validation was performed on the data acquired using our clinical real-time platform [23] interfaced with the Epic^®^ EHR system.

### 2.2 Ethics Approval and Patient Consent

Retrospective data from UFH (UFH-R) was obtained with the approval of the University of Florida Institutional Review Board and Privacy Office as an exempt study with a waiver of informed consent (IRB201901123). Prospective data from UFH (UFH-P) was obtained by consent under the approval of the University of Florida Institutional Review Board under the numbers 201900354 and 202101013. Before enrolling patients in the study, written informed consent was obtained from all participants. In cases where patients could not provide informed consent, consent was obtained from a legally authorized representative (LAR) acting on their behalf. Eligible participants were individuals aged 18 and older who were admitted to the ICU and expected to remain there for at least 24 hours. Patients who could not provide an LAR or self-consent were expected to be transferred or discharged from the ICU within 24 hours, and those necessitating contact or isolation precautions were excluded. Also excluded from this study were patients who expired within 24 hours of recruitment. The eICU dataset is deidentified and publicly available. The data in the MIMIC dataset is de-identified, and the institutional review boards of the Massachusetts Institute of Technology and Beth Israel Deaconess Medical Center both approved using the database for research.

### 2.3 Features and Outcomes

**Fig. 2 |:**
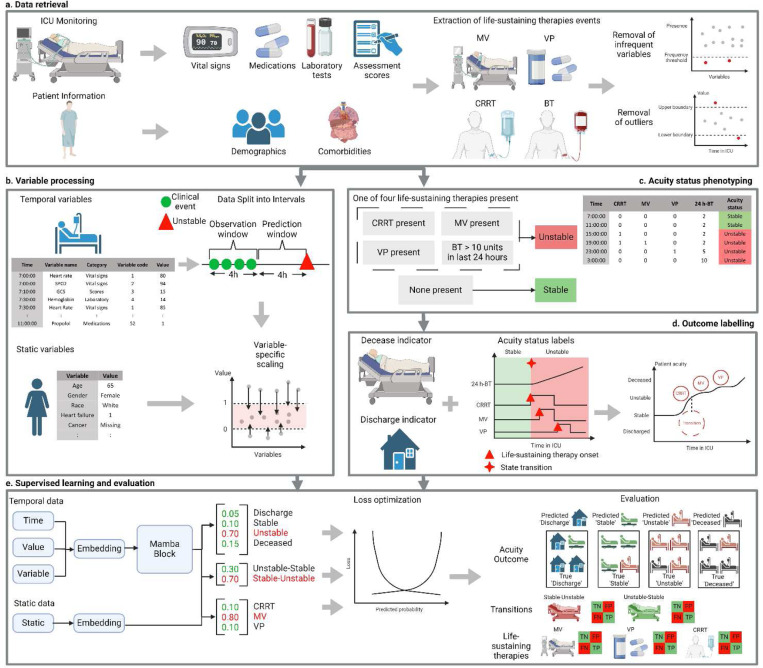
APRICOT-M model development overview. **(a)** Data from three different Intensive Care Unit (ICU) datasets were used for training and validating the APRICOT-M model (199 hospitals, 195,516 patients, and 259,706 ICU admissions total). Each dataset consisted of ICU stay information (*i.e.*, vital signs, medications, laboratory test results, and assessment scores) and admission information (*i.e.*, patient demographics and comorbidities). The presence of four life-sustaining therapies events were extracted: mechanical ventilation (MV), vasopressors (VP), continuous renal replacement therapy (CRRT), and massive blood transfusion (BT). Highly infrequent and outlier features were removed. **(b)** Temporal variables were processed into a list of clinical events for each patient, where each variable was assigned a code according to its order of appearance. Clinical events were assigned to 4-hour observation windows and used with static patient information to predict the outcome in the next 4-hour window. After data was split into time windows, each variable was scaled. **(c)** The acuity status was determined every 4 hours in the ICU, with a patient being labeled as ‘unstable’ for a 4-hour interval if at least one of the four life-sustaining therapies extracted was present. **(d)** The outcome for each 4-hour window was determined by combining acuity status labels with decease/discharge indicators, providing a complete acuity trajectory for each patient, including the transition between states and onset of three life-sustaining therapies: MV, VP, and CRRT. **(e)** A state space-based model was employed for acuity, transition, and life-sustaining therapy onset prediction, with two separate embeddings used for temporal and static data. The first embedding was created by a Mamba block, which took the sequence of clinical events, from which the output was then combined with the second embedding, created from static patient data, to calculate the probabilities of each acuity outcome, transition and life-sustaining therapy. The loss of the model was optimized by comparing the predicted probabilities with the ground truth labels. Finally, the model was evaluated by calculating the classification error for each outcome individually.

The primary outcome predicted by APRICOT-M is the acuity state (obtained using computable phenotyping) of a patient in the ICU in the next four hours defined by four levels of severity from the least to the most severe: discharge, stable, unstable, and deceased [12]. Four-hour windows were chosen to capture the continuous transitions between acuity states that can happen in short time periods [12]. Discharge and deceased states were determined through indicators of whether a patient was discharged or transferred out of the ICU alive or passed away in the ICU (Fig. 2d). Unstable and stable were determined by the presence of one of four life-sustaining therapies. Specifically, if a patient was receiving continuous renal replacement therapy (CRRT), on intravenous vasopressors (VP) (either continuous infusion or one-time push), on invasive mechanical ventilation (MV), or had a massive blood transfusion (>10 units in the last 24 hours) (BT), the patient was categorized as unstable for the time intervals during which any of these therapies were present (Fig. 2c). Otherwise, the patient was considered stable.

Transitions to instability (*i.e.*, Stable-Unstable) and to stability (*i.e.*, Unstable-Stable) were labeled as outcomes to address the potential bias of predicting the current state (*e.g.*, patient which is currently stable, and the model predicts the next state to be stable as well). The onset of CRRT, MV, and VP were also labeled to predict the potential need for one or more of these therapies (Fig. 2d). In the case of BT, prediction could not be performed, given the label was defined by 24-hour periods and the patient would have undergone the therapy by the time of prediction.

In order to predict the outcome for the next four hours, static patient information and clinical events from the previous four hours were used as predictive features. Clinical events consisted of the timestamp converted to hours since admission, a variable code, and the value of the measured variable. Simultaneously occurring features were arbitrarily ordered. Events were assigned to their corresponding observation window, where an observation window was comprised of all clinical events in the 4 hours prior to the start of the current prediction window. The prediction window represents a four-hour interval in which the outcome is assessed (Fig. 2b). In particular, vital signs, medications, laboratory tests, and assessment scores were extracted from the previous four hours of ICU monitoring, while demographic and comorbidity information were extracted from patient history information. Variables occurring in less than 5% of all ICU stays were removed to prevent the absence of variables in the training set that could have been present in the validation sets. Outliers were also removed based on impossible values based on clinical expertise and based on an upper (99th percentile) and lower (1st percentile) bound (Fig. 2a). A complete list of the variables used for prediction can be found in the Supplementary Material (Supplementary Table S1). Both temporal and static variables were then scaled according to their specific range using minimum-maximum scaling based on the minimum and maximum values for each feature in the development cohort (Fig. 2b). Finally, the APRICOT-M model, which uses the embedding schema of a clinical time-series model [19] with incorporation of a state-space model (Mamba block, details in section 2.4) [20], was trained to predict probabilities for each acuity outcome, transition, and life-sustaining therapy onset and evaluated against ground truth labels (Fig. 2e).

### 2.4 Model Development and Performance

**Fig. 3 |:**
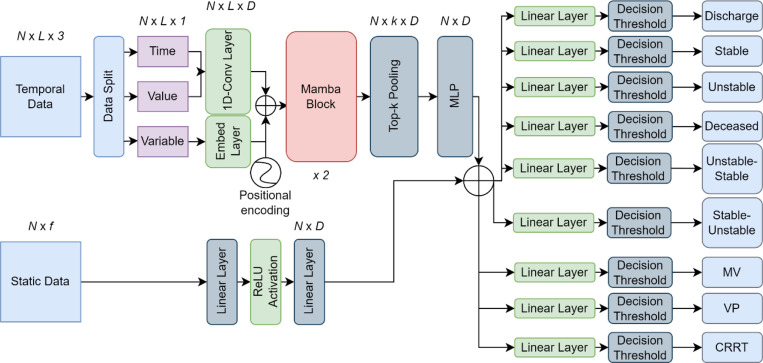
APRICOT-M model architecture. State space model adapted to a clinical setting. The temporal data of dimensions *N* (number of samples) × *L* (sequence length) × 3 (triplet recording for each event) was split into individual matrices for time, value, and variable code of dimensions *N* × *L* each. The time and value matrices went through a 1D-Convolutional Layer to create one embedding, while the variable code matrix went through a look-up embedding layer to retrieve the embedding for the specific variable. Both embeddings were fused, and a positional encoding was added to conserve the sequence order information. The fused embedding was passed through two Mamba blocks, from which top-*k* pooling was applied to retrieve the *k*-most important states. The vector was then passed through a multi-layer perceptron (MLP) to create an *N* × *D* embedding fused with the static embedding resulting from passing the static data of dimensions *N* × *f* (static features) through two linear layers. Finally, the resulting vector was passed through four linear layers, which yield the probability for each acuity primary outcome (discharge, stable, unstable, decease), from which a decision threshold is applied to each outcome. The vector was also passed through two and three linear layers along with the application of a decision threshold to determine state transition and need for life-sustaining therapies (CRRT, MV, VP), respectively. The acuity status for the next four hours was determined by applying a logic schema.

After splitting the three datasets into development, external, temporal, and prospective, the development dataset was further split by randomly selecting 80% of the patients for training and 20% for validation and tuning hyperparameters. The APRICOT-M model was developed based on the embedding schema of a clinical Transformer architecture [19], incorporating a Mamba block [20]. The original architecture was modified to create an embedding for temporal data using a sequence of triplets of time, value, and variable code recordings. The time and value recordings were passed through one-dimensional (1D)-Convolutional layers, and the variable code recordings through an Embedding layer. The results were fused as one temporal embedding through addition, and positional encoding was incorporated to preserve the sequence order information. This approach removed the need for imputation that other time-series models require to maintain fixed dimensions in the input. The embedding schema presented here used padding/truncation to match the maximum sequence length set for the model, so no feature values were imputed. For static data, an embedding was created using two linear layers. A Mamba block was then employed to add context to the temporal embedding, from which the resulting vector was fused (*i.e.*, added) with the static embedding. Finally, the fused vector was passed through individual linear layers to calculate the probability of each acuity primary outcome, transition, and life-sustaining therapy (Fig. 3).

**Fig. 4 |:**
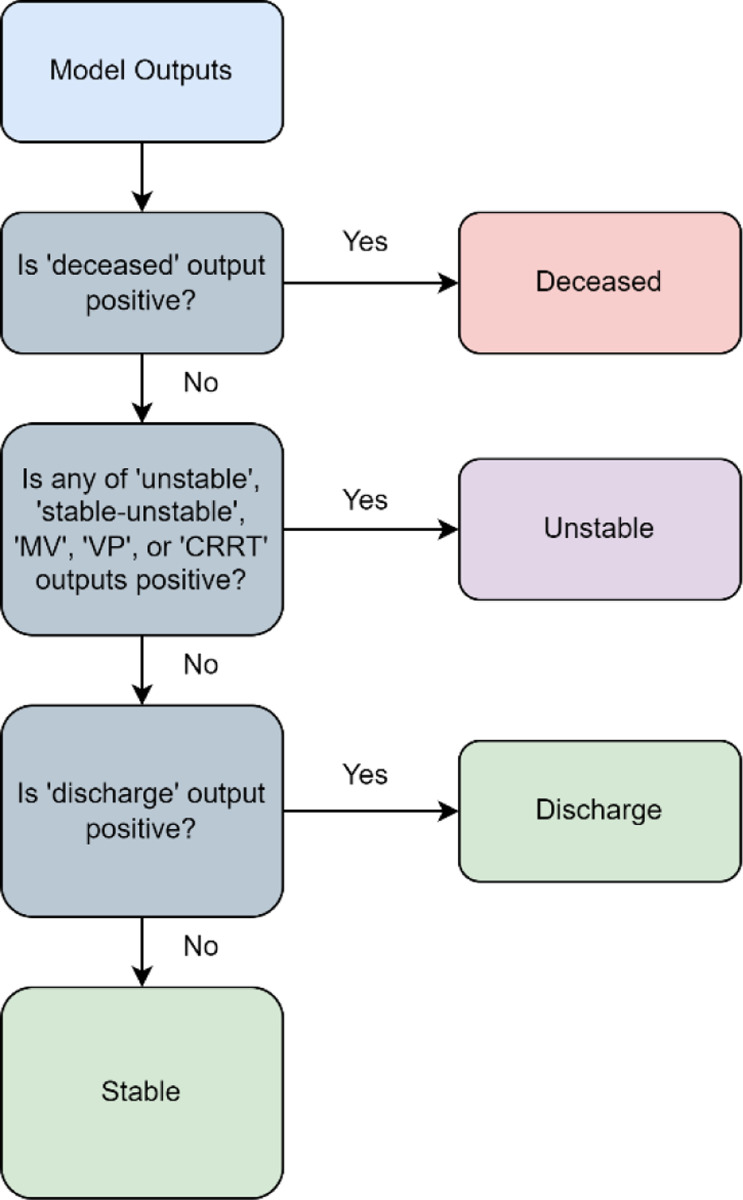
Prediction logic. The model outputs were processed using a prediction logic to determine the acuity status. The deceased state was determined through the presence of a positive output for the ‘deceased’ prediction head and is given priority, given that it is the most severe state. The unstable state was determined if the ‘deceased’ output was negative and through the presence of a positive label for any of the ‘unstable’, ‘stable-unstable’, ‘MV’, ‘VP’, or ‘CRRT’ prediction heads. The presence of a positive label for any of the life-sustaining therapies (MV, VP, or CRRT) would indicate an unstable status. The discharge state was determined if both ‘deceased’ and ‘unstable’ outputs were negative and if the ‘discharge’ output is positive. Finally, a stable state was determined if all other three states were negative.

For training the model, class weights were applied to the loss function to account for the class imbalance. Four algorithms were used as baseline models for comparison: categorical boosting (CatBoost) [25], gated recurrent unit (GRU) [26], a vanilla Transformer [16], and APRICOT-T. For CatBoost, statistical features (*i.e.*, missingness indicators, minimum, maximum, mean, and standard deviation) were extracted to convert temporal data into a static representation. For the GRU and vanilla Transformer, temporal features were resampled to 20-minute resolution and tabularized to have the same sequence length and dimensions. For APRICOT-T, the same data processing as APRICOT-M was applied as well as the same architecture with the only difference of replacing the Mamba blocks with Transformer encoder layers. A logic schema (shown in Fig. 4) was implemented in the model outputs to predict the overall acuity status. For evaluation, six metrics were used: the Area Under the Receiver Operating Characteristic (AUROC), the Area Under the Precision-Recall Curve (AUPRC), sensitivity, specificity, Positive Predictive Value (PPV), and Negative Predictive Value (NPV). The Youden index [27] was used to find the optimal threshold to calculate sensitivity, specificity, PPV and NPV. Both AUROC and AUPRC were used as metrics to tune the model architecture and hyperparameters on the development test.

The Sequential Organ Failure Assessment (SOFA) [4] score was used as a clinical mortality and instability prediction baseline. For mortality, the raw SOFA score was scaled to a 0–1 range [28] and directly evaluated using AUROC and AUPRC. For instability, two approaches were used: scaling raw SOFA score to 0–1 range and a criterion of 2 or more points increase from baseline SOFA (*i.e.*, the SOFA score at admission) to label 4-hour windows as unstable based on previous literature using this criterion to indicate increased mortality risk [29]. Both approaches were evaluated as well using AUROC and AUPRC.

### 2.5 Calibration and Subgroup Analysis

Due to differences in the outcomes incidences (*i.e.*, positive rates) in the four study cohorts, APRICOT-M was calibrated using an isotonic regression algorithm [30] to improve the accuracy of the risk probability predictions. A random sampling of 10% for each validation set was used for model calibration. Calibration performance was evaluated by the Brier score and calibration curve. We evaluated model performance by comparing important demographic subgroups in all cohorts, including sex (male and female), age (young [18–60] and old [over 60]), and race (Black, White, and Others).

### 2.6 Acuity Episode Prediction and False Positive Analysis

**Fig. 5 |:**
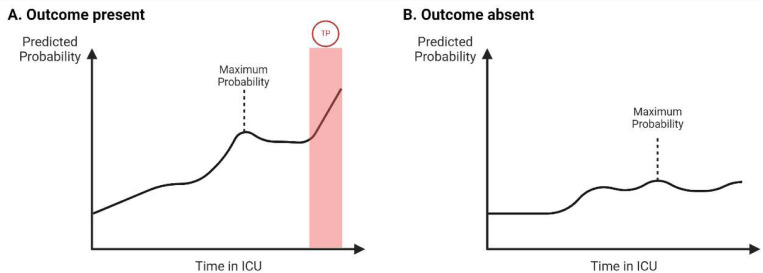
Episode level prediction. (A) Calculation of episode level prediction using maximum predicted probability across all 4-hour windows in ICU admission before the outcome onset when the outcome was present. (B) Calculation of episode level prediction using maximum predicted probability across all 4-hour windows during ICU admission when the outcome was absent.

Given that the prediction problem is a continuous prediction problem, evaluating the model at the step level (*i.e.*, 4-hour window level) does not account for the possibility of the model making true positive predictions earlier than expected (*i.e.*, predicting an outcome more than 4 hours before). In other words, the model could predict the occurrence of an outcome rather than the exact time when it will happen. Therefore, both step-level and episode-level (*i.e.*, outcome occurrence) performance were compared for mortality and instability outcomes. For the episode-level performance, both AUROC and AUPRC were computed using the maximum predicted probability of each outcome across all 4-hour windows for each ICU admission when the outcome was present or absent (Fig. 5). Sensitivity, specificity, and PPV were computed both at step and episode levels for different thresholds according to desired episode level precision.

### 2.7 Time Window Analysis

To determine the performance between different time window lengths, the same methodology used at the 4-hour window level was applied to 24-hour and 48-hour observation and prediction time windows. Given the necessity of longer ICU admissions to allow at least one prediction for each admission at 24-hour and 48-hour windows, the cohort sizes for these analyses were smaller. Cohort diagrams for both 24-hour and 48-hour time window cohorts are provided in the Supplementary Material (Supplementary Figures S1 and S2).

### 2.8 Model Interpretability

For the interpretation of the APRICOT-M model outputs, the integrated gradients method was used [31]. Integrated gradient attributions were calculated for each temporal variable in the embedding layer as well as for each static variable in the static embedding. This method allowed computation of the importance of a feature and the time step, obtaining an overall score for each input feature to better understand risk predictors for each outcome.

### 2.9 Statistical Analysis

To determine if the difference in performance between baseline models and APRICOT-M was statistically significant, all metric values between algorithms were compared using a Wilcoxon rank sum test. A 100-iteration bootstrap was performed to calculate the 95% confidence interval (CI) for each performance metric, and the median across the bootstrap was used to represent the overall value of each metric.

## 3. Results

### 3.1 Patient Characteristics

Patient characteristics for this study are presented in Table 1 in terms of ICU admissions. The external cohort had the highest median age (65 years), while the prospective cohort had the lowest (60 years). There were no significant differences in the percentage of female patients between the development, external, and temporal cohorts, but there were fewer in the prospective cohort (40.1%) compared to the development and external cohorts. The prospective cohort had the highest ICU length of stay (6.1 days), while the external cohort had the lowest (1.9 days). The incidence of mechanical ventilation was significantly higher in the external cohort (34.2%) compared to the development and temporal cohorts. The incidence of vasopressors was highest in the temporal cohort (28.8%) compared to all other cohorts. The temporal and external cohorts had the highest mortality rate (5.9% and 5.8%, respectively), while the prospective cohort had the lowest (2.7%). Comparisons between patients who did not experience instability during their ICU stay and survived against patients that received MV, VP, and CRRT therapies and deceased patients are provided in the Supplement for all cohorts (Supplementary Tables S2-S5).

### 3.2 State Transitions

**Fig. 6 |:**
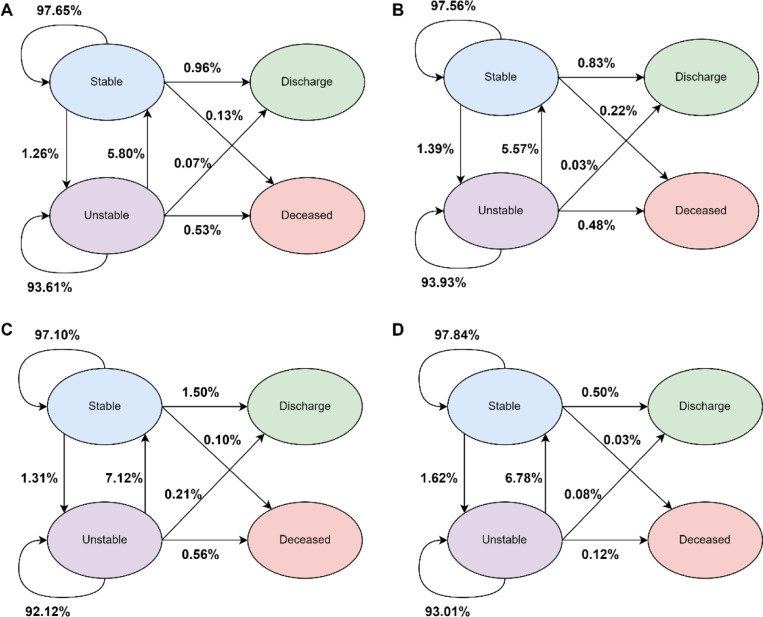
State transition probability. (A) Development cohort. (B) External cohort. (C) Temporal cohort. (D) Prospective cohort. The acuity state transition probability is shown for every 4-h window. The probability was calculated based on the patient’s current state (either stable or unstable) and the probability to transition to each acuity state. The probabilities for discharge and death were not calculated since they are end outcomes.

The probability for each state transition across all study cohorts was calculated and shown in Fig. 6. For stable patients, there was a 97.10–97.84% probability of remaining stable, 1.26–1.62% of transitioning to an unstable state, 0.50–1.50% of transitioning to discharge, and 0.03–0.22% of transitioning to deceased. For unstable patients, there was a 92.12–93.93% probability of remaining unstable, 5.57–7.12% of transitioning to stable, 0.03–0.21% of transitioning to discharge, and 0.12–0.56% of transitioning to deceased.

### 3.2 Model Performance, Calibration, and Subgroup Analysis

The AUROC performance of APRICOT-M to predict outcomes within the next 4 hours was calculated for development, external, and prospective cohorts (Table 2). Results for the temporal cohort can be found in the Supplementary Material. Performance for discharge prediction did not show significant differences among development, external, and prospective cohorts. Prediction of the deceased state had the highest performance in the prospective cohort (0.99 CI: 0.96–1.00) and lowest performance in the development cohort (0.94 CI: 0.94–0.95), while prediction of stable-unstable transition had higher performance in the development and external cohort (0.77 CI: 0.76–0.77, and 0.77 CI: 0.77–0.77 respectively) compared to the prospective cohort (0.71 CI: 0.67–0.75). Prediction for life-sustaining therapies showed highest performance on development and external cohorts for MV (0.80 CI: 0.79–0.81, and 0.81 CI: 0.80–0.81 respectively), on development and prospective cohorts for VP (0.81 CI: 0.80–0.82, and 0.83 CI: 0.78–0.87), and development cohort for CRRT (0.94 CI: 0.93–0.96). All other metrics and comparisons to baseline models are shown in Supplementary Table S6. Calibration curves and Brier scores for each cohort are shown in Supplementary Figures S3-S6. Subgroup analysis based on age, gender, and race groups is shown in Supplementary Table S13. Given the small size of the prospective cohort and the low incidence rate of certain outcomes, some age, sex, and race groups did not have positive labels for some outcomes (*e.g.*, no positive labels for mortality or transitions). Therefore, the performance metrics could not be computed for these groups and the subgroup bias analysis could not be performed.

### 3.3 Acuity Episode Prediction and False Positive Analysis

The performance of APRICOT-M at both step and episode levels for mortality and instability predictions is summarized in Tables 3 and 4. For mortality, APRICOT-M achieved higher AUROC and AUPRC for episode and step-level prediction across the development, external, and prospective cohorts compared to the raw SOFA score, with all differences being statistically significant except episode AUROC for the prospective cohort. For instability, APRICOT-M was compared to the raw SOFA score and the two or more-point increase criterion. For development and external cohorts, APRICOT-M had higher AUROC at both episode and step levels and higher AUPRC at the episode level than raw SOFA scores and SOFA (≥ 2 points). For the prospective cohort, APRICOT-M had no statistically significant differences at the episode level with both SOFA models but had higher AUROC at the step level.

The prediction metrics for APRICOT-M compared to SOFA at different episode-level precisions (*i.e.*, the PPV of the model for episode predictions) for mortality and instability are shown in Supplementary Tables S7 and S8 respectively. When using a 33% precision at the episode level, APRICOT-M predicted 76%, 80%, and 88% of deceased patients in the development, external, and prospective cohorts, respectively, compared to 44%, 34%, and 73% predicted by SOFA. For instability, different precision levels for SOFA could not be calculated, given the low AUPRC. Therefore, the SOFA (≥ 2 points) criterion was used as the baseline. At 33%-episode precision, APRICOT-M predicted 36%, 55%, and 51% of instability cases compared to 36% (at 12% precision), 24% (at 11% precision), and 37% (at 27% precision) predicted by SOFA in the development, external, and prospective cohorts respectively.

The metrics at both episode and step levels at 33% precision for mortality, instability, MV, and VP prediction are shown in Table 5. This precision level was chosen given it has been used on previous clinical continuous risk prediction studies [32] and yielded the best balance between the number of false positives and sensitivity. The earliest prediction (*i.e.*, lead time) was measured as the number of hours before the onset of the outcome at which the first positive prediction was made, and the number of alerts represents the number of times the model predicted a positive before the outcome. For mortality, 76–88% of cases were correctly predicted with 2–3 alerts and up to 20–200 hours of lead time. For instability, 36–55% of cases were correctly predicted with 1–2 alerts and up to 4–14 hours of lead time. For MV, 44–63% of cases were correctly predicted with 1–2 alerts and up to 4–18 hours of lead time. For VP, 27–51% of cases were correctly predicted with one alert and up to 4 hours of lead time. The metrics for all models at episode and step level for different thresholds for mortality, instability, MV and VP are shown in Supplementary Tables S9-S12.

### 3.4 Time Window Analysis

The performance of APRICOT-M across different time windows is shown in Table 6. The best performance for most outcomes was achieved with a 4-h time window with statistical significance, except for Unstable-Stable prediction, which had the best performance with a 24-h time window in the development and external cohorts, and CRRT, which also had the best performance with a 24-h time window in the external cohort.

### 3.5 Predictions distribution

**Fig. 7 |:**
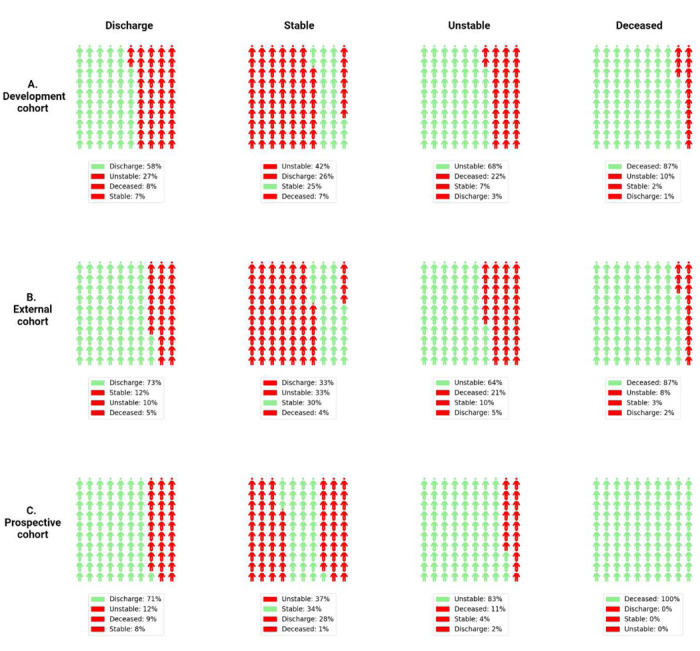
Predicted proportions of acuity status at step level. (A) Development cohort. (B) External cohort. (C) Prospective cohort. Each plot shows the proportion of predicted acuity states for each state according to the column: discharge, stable, unstable, and deceased. Green represents a correct prediction (*i.e.*, true positives) for those with the specific acuity state, while red represents an incorrect prediction (*i.e.*, any state other than the correct one was predicted).

**Fig. 8 |:**
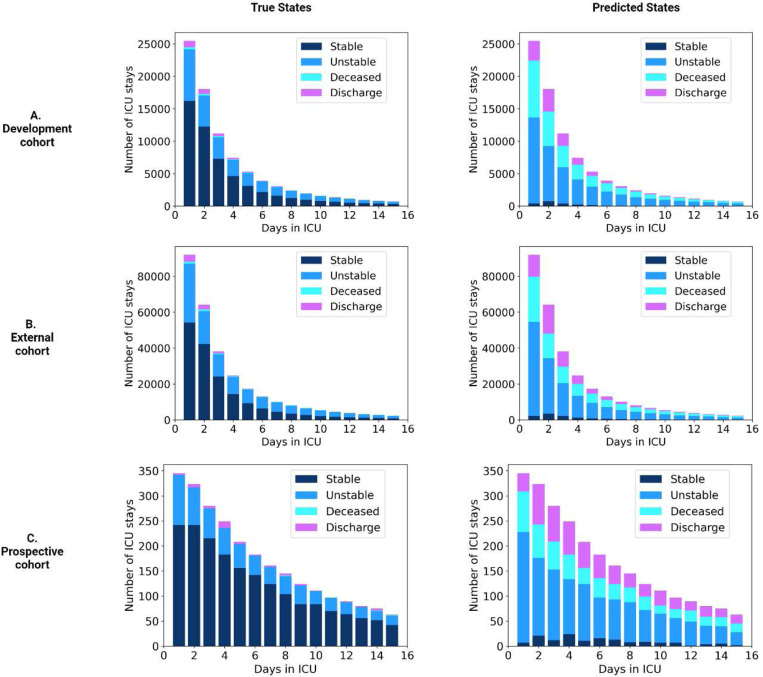
Distribution of true and predicted acuity states across days in ICU. (A) Development cohort. (B) External cohort. (C) Prospective cohort. The left side shows the true distribution of acuity states in the first 15 days of ICU admission for each cohort, while the right side shows the distribution of predicted acuity states in comparison.

To further evaluate the performance of APRICOT-M in acuity prediction, a confusion matrix was generated for the four primary outcomes at the step (*i.e.*, 4-h window) level: discharge, stable, unstable, and deceased. The proportions of predicted acuity status (Fig. 7) show that across the development, external, and prospective cohorts, the sensitivity for predicting discharge and mortality ranged between 58–74% and 87–100%, respectively. The sensitivity range for predicting stable and unstable states was 25–37% and 64–74%, respectively, with 26–33% of stability cases predicted as discharge and 11–22% predicted as deceased. Furthermore, the proportions of each acuity state across each day in the ICU are shown in Fig. 8 compared to the predictions by APRICOT-M. This figure shows more discharge, unstable, and deceased state predictions than the ground truth number of these states.

### 3.6 Feature importance

**Fig. 9 |:**
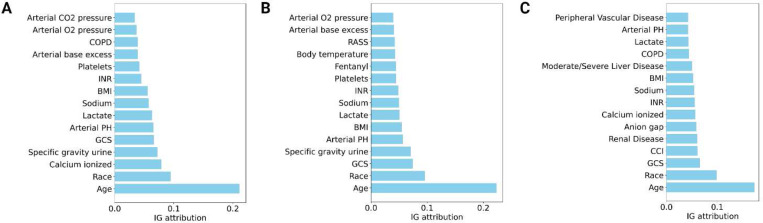
Feature importance. (A) Development cohort. (B) External cohort. (C) Prospective cohort. The top 15 features for primary outcome prediction. Features are ordered according to the highest average of integrated gradient attributions across the increased acuity outcomes: unstable, stable-unstable, MV, VP, CRRT, and deceased.

The relevance of each feature for the prediction of increased acuity was determined using integrated gradients. The integrated gradients of each increased acuity outcome (*i.e.*, unstable, stable-unstable, MV, VP, CRRT, and deceased) were averaged to obtain the overall score for each feature that drives the model to predict higher acuity level. The top 15 features across all development, external, and prospective cohorts are presented in Fig. 9. The top features among all cohorts were age, race, Glasgow coma scale (GCS) score, lactate, sodium, and INR (blood clot). A complete feature ranking for all prediction tasks across all cohorts is provided in the Supplement (Supplementary Figures S7-S10).

### 3.7 Examples of Real-Time Evaluation

**Fig. 10 |:**
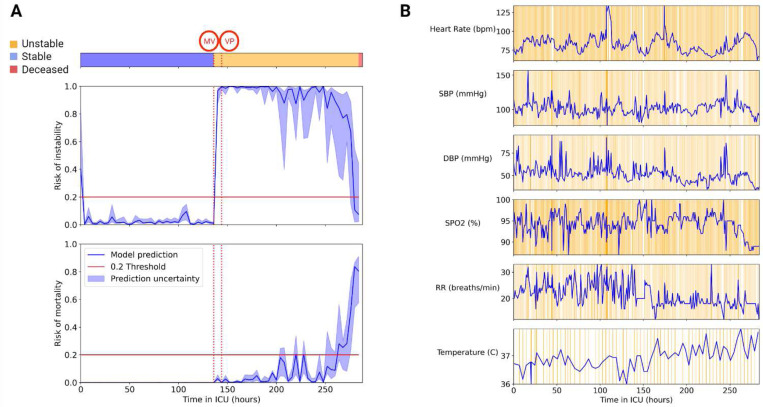
APRICOT-M monitoring, deceased patient. Example of real-time monitoring of a deceased patient in the external cohort. (A) Acuity trajectory of the patient, with the onset of vasopressor (VP) and mechanical ventilation (MV) therapies, along with instability and mortality risk predicted by APRICOT-M throughout ICU admission. (B) Vitals trajectory of the patient including Heart Rate (HR), Diastolic Blood Pressure (DBP), Systolic Blood Pressure (SBP), Respiratory Rate (RR), Oxygen Saturation (SPO2), and Body Temperature. Orange shading represents integrated gradient attributions at each time step, with higher color intensity representing a higher attribution.

**Fig. 11 |:**
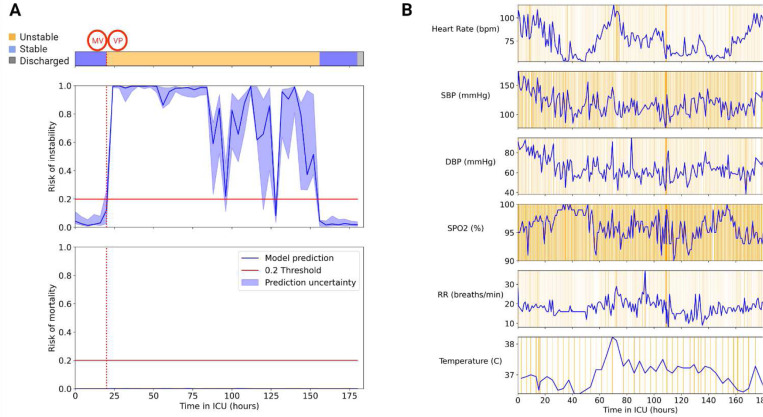
APRICOT-M monitoring, discharged patient. Example of real-time monitoring of a discharged patient in the external cohort. (A) Acuity trajectory of the patient, with the onset of vasopressor (VP) and mechanical ventilation (MV) therapies, along with instability and mortality risk predicted by APRICOT-M throughout ICU admission. (B) Vitals trajectory of the patient, including Heart Rate (HR), Diastolic Blood Pressure (DBP), Systolic Blood Pressure (SBP), Respiratory Rate (RR), Oxygen Saturation (SPO2), and Body Temperature. Orange shading represents integrated gradient attributions at each time step, with higher color intensity representing a higher attribution.

Two example patients were extracted from the external cohort to demonstrate APRICOT-M real-time capabilities. The first example was a patient who passed away. This patient transitioned from stable to unstable approximately 136 hours after ICU admission when they required mechanical ventilation (MV). About 8 hours later, the patient received vasopressor (VP) medication and ultimately passed away approximately 280 hours after ICU admission. The APRICOT-M model detected increased mortality risk at around 200 hours (Fig. 10A), increasing until death. The vitals chart in Fig. 10B show the integrated gradient attribution to each time step for each vital sign along the patient’s trajectory in the ICU. The second example was a patient discharged to their home at the end of their ICU stay. This patient transitioned from stable to unstable approximately 20 hours into their ICU admission when they simultaneously received MV and VP therapies. The patient remained unstable until about 156 hours into their admission when they transitioned back to stable and was discharged 24 hours later. The APRICOT-M model detected decreased instability risk approximately 16 hours before the patient transitioned to a stable condition (Fig. 11A). Also, mortality risk remained low at all times during the ICU stay.

## 4. Discussion

The results presented in this study show the development and validation of APRICOT-M as a tool for ICU patient acuity monitoring. To the best of our knowledge, this is the first study to predict acuity in the ICU based on life-sustaining therapies (MV, VP, CRRT, and BT). Furthermore, discharge and mortality data were integrated to augment patient outcome and discharge disposition predictions. Previous studies to predict patient acuity in the ICU have focused on mortality prediction as a proxy of acuity [9], [10]. Comparing mortality prediction in this study in terms of AUROC, performance on development (0.94 CI: 0.94–0.95), external (0.95 CI: 0.94–0.95), and prospective (0.99 CI: 0.96–1.00) cohorts exceeds DeepSOFA (AUROC 0.90–0.91) [9] and is comparable to the Transformer acuity estimation model (AUROC 0.98) [10]. Furthermore, this study has the advantage of validating mortality prediction performance on patients from multiple hospitals, which demonstrates the generalizability of APRICOT-M.

The model presented here has the ability to predict the type of life-sustaining therapy that the patient will need in addition to overall acuity predictions. Although no studies have predicted the need for CRRT to the best of our knowledge, some studies have predicted the use of VP and MV. For VP prediction, the AUROC in development and prospective sets (0.81 CI: 0.80–0.82 and 0.83 CI: 0.78–0.87) was higher than what is seen in the literature (AUROC 0.80–0.81) [33], while the external set showed lower, although comparable, performance (0.76 CI: 0.76–0.77). For MV prediction, performance reported in the literature is varied, with some studies reporting as low as AUROC 0.68 [34] and others reporting as high as AUROC 0.97 [35]. It is important to note that these studies performed MV prediction on COVID-19 patients which could be a potential bias. Furthermore, the models were developed and validated on data from only one hospital. The diverse data collection methods of each hospital can make it difficult to extract with high precision the start and end times of these events. The AUROC shown in this study falls within the range of what is seen in the literature (development 0.80 CI: 0.79–0.81, external 0.81 CI: 0.80–0.82, and prospective 0.73 CI: 0.69–0.77) and is tested in data from multiple hospitals. The lower performance in ventilation prediction in the prospective cohort could be explained by the lower acuity level of patients in this cohort, given this cohort was the only one where patients were consented. Patients with lower acuity are more likely to agree to participate in the study since they have are experiencing fewer health issues.

To evaluate the real-world performance of APRICOT-M, evaluating performance at the episode level was fundamental to account for correct prediction of occurrence of an outcome (*i.e.*, episode level) rather than correct prediction of the time where the outcome would occur (*i.e.*, step level). By setting a threshold at the episode level, the model can achieve higher precision at the episode and step level. The APRICOT-M model demonstrated statistically significant better performance at both episode and step levels compared to SOFA scores for mortality and instability predictions and predicted more mortality and instability cases at the 33% precision episode level. This precision level means that when APRICOT-M predicts a positive outcome, 1 out of 3 times, the outcome will occur in the next four or more hours.

The distribution of predictions shown in Fig. 7 demonstrates that APRICOT-M correctly identifies the primary outcomes with high sensitivity for deceased (87–100%), unstable (64–83%), and discharged (58–73%). Although sensitivity for stability (25–34%) was lower, many false negatives were predicted as discharged (26–33%), which could indicate the patients were in a condition where they could be discharged at the moment but were discharged later. These numbers also reflect the high count of false positive alarms for unstable states in stable 4-hour windows (33–42%). This is also reflected in Fig. 8, where a significant proportion of the model’s output is unstable predicted labels. As previously shown in the episode prediction, the model often alerts several hours before a patient transitions to an unstable state, which inflates the number of false positives.

The two examples presented in Fig. 10 and Fig. 11 show the potential use of APRICOT-M in real-time in the ICU. For the deceased patient example, the model recognized increased mortality risk several days before the patient passed away. On the other hand, the model recognized decreased instability risk similarly for the discharged patient example. These two examples show the capability of APRICOT-M to provide early warnings for more timely interventions.

The use of integrated gradients identified several features which could be important for acuity prediction. The importance of age for this type of prediction is consistent with the knowledge that older patients are at a higher risk in the ICU [36]. The GCS score, used to determine comatose state, is also correlated to patient acuity [37]. Lactate, sodium, and INR (blood clot) have all been shown to be correlated to higher ICU mortality and therefore higher levels of acuity [38], [39], [40]. The high attribution given to race by integrated gradients could be due to different factors including social determinants of health. In fact, some studies have shown the impact of these factors in readmissions across a variety of conditions [41], [42]. Future studies will explore how and why race is associated with patient acuity in the ICU.

The prospective evaluation of APRICOT-M on ICU patient data acquired with our real-time platform provides a validation method of the potential application of this model in the real world. The performance seen in this cohort was comparable to the other three cohorts, which shows APRICOT-M can be readily deployed and employed in real-time to assist clinicians in decision-making. However, deployment of the model in real-time and larger prospective cohorts is necessary to validate the model’s real-time performance fully.

Some of the limitations of this study include the challenge of labeling outcomes in different hospital settings. Given the high number of hospitals in this study, labeling procedures such as MV and CRRT can be inconsistent, due to potential restrictions from some hospitals to access this data. Therefore, the datasets could be incomplete, and therefore, the performance of the model is limited. The small number of features can limit model performance as well. Large models can usually benefit from a large number of features. The present study used a limited subset of features in all three databases (UFH, MIMIC, and eICU). Since these features were manually mapped between datasets, some features could have been missed. Future studies will employ the Observational Medical Outcomes Partnership (OMOP) Common Data Model to map features more effectively and accurately between datasets.

In summary, we have developed and validated both retrospectively and prospectively APRICOT-M, a state space model to predict patient acuity in the ICU. This tool allows for real-time acuity monitoring of a patient and can provide helpful information to clinicians to make timely interventions. Furthermore, the model can suggest life-sustaining therapies the patient might need.

## Figures and Tables

**Table 1.** Baseline characteristics for ICU admissions of four study cohorts.

Item	Development (*n* = 142,062)	External (*n* = 101,356)	Temporal (*n* = 15,940)	Prospective (*n* = 369)

**Basic information**				
Number of patients	107,473	74,901	12,927	215
Number of hospital encounters	129,055	93,007	15,940	326
Age, years, median (IQR)	64.0 (52.0–75.0) ^b,c,d^	65.0 (53.0–76.0) ^a,c,d^	62.0 (49.0–72.0) ^a,b^	60.0 (48.0–68.0) ^a,b^
Female, n (%)	64,485 (45.4%) ^d^	46,031 (45.4%) ^d^	7,158 (44.9%)	148 (40.1%) ^a,b^
BMI, kg/m^2^, median (IQR)	27.5 (23.7–32.5) ^c,d^	27.5 (26.0–29.0) ^c,d^	27.5 (23.3–32.3) ^a,b,d^	26.6 (23.0–30.5) ^a,b,c^
ICU length of stay, days, median (IQR)	2.0 (1.1–3.8) ^b,c,d^	1.9 (1.1–3.5) ^a,c,d^	3.1 (1.7–6.2) ^a,b,d^	6.1 (3.1–12.1) ^a,b,c^
CCI, median (IQR)	0.0 (0.0–1.0) ^b,c,d^	0.0 (0.0–0.0) ^a,c,d^	2.0 (1.0–4.0) ^a,b,d^	2.0 (1.0–4.0) ^a,b,c^
**Race, n (%)**				
Black	20,027 (14.1%) ^b,c^	10,435 (10.3%) ^a,c,d^	3,046 (19.1%) ^a,b,d^	53 (14.4%) ^b,c^
White	109,382 (77.0%) ^b,c^	72,512 (71.5%) ^a,c,d^	11,881 (74.5%) ^a,b^	286 (77.5%) ^b^
Other	12,653 (8.9%) ^b,c^	18,409 (18.2%) ^a,c,d^	1,013 (6.4%) ^a,b^	30 (8.1%) ^b^
**Comorbidities, n (%)**				
Congestive heart failure	12,304 (8.7%) ^b,c,d^	6,979 (6.9%) ^a,c,d^	4,806 (30.2%) ^a,b,d^	88 (23.8%) ^a,b,c^
Chronic obstructive pulmonary disease	13,566 (9.5%) ^b,c,d^	6,824 (6.7%) ^a,c,d^	4,761 (29.9%) ^a,b,d^	73 (19.8%) ^a,b,c^
Renal disease	10,044 (7.1%) ^b,c,d^	8,747 (8.6%) ^a,c,d^	3,577 (22.4%) ^a,b,d^	109 (29.5%) ^a,b,c^
**Life-sustaining therapies, n (%)**				
MV	47,533 (33.5%) ^b,c^	34,701 (34.2%) ^a,c^	4,595 (28.8%) ^a,b,d^	132 (35.8%) ^c^
VP	26,068 (18.3%) ^b,c,d^	24,688 (24.4%) ^a,c,d^	4,586 (28.8%) ^a,b,d^	70 (19.0%) ^b,c^
CRRT	1,535 (1.1%) ^b,c^	1,662 (1.6%) ^a,c^	392 (2.5%) ^a,b^	4 (1.1%)
BT	279 (0.2%)	21 (0.0%)	186 (1.2%)	0 (0.0%)
**Outcomes, n (%)**				
Mortality	7,391 (5.2%) ^b,c,d^	5,916 (5.8%) ^a,d^	942 (5.9%) ^a,d^	10 (2.7%) ^a,b,c^

Abbreviations: BMI: Body Mass Index; BT: Massive Blood Transfusion; CCI: Charlson Comorbidity Index; CRRT: Continuous Renal Replacement Therapy; IQR: interquartile range; MV: Mechanical Ventilation; VP: Vasopressors.

P-values for continuous variables are based on a pairwise Wilcoxon rank sum test. P-values for categorical variables are based on pairwise Pearson’s chi-squared test for proportions.

a p-value < 0.05 compared to the development cohort.

b p-value < 0.05 compared to external cohort.

c p-value < 0.05 compared to temporal cohort.

d p-value < 0.05 compared to prospective cohort.

**Table 2.** APRICOT-M performance for 4-h predictions in terms of AUROC for three study cohorts.

Outcomes	Development	External	Prospective

Primary			
Discharge	0.85 (0.85–0.86)	0.85 (0.85–0.85)	0.86 (0.82–0.90)
Stable	0.91 (0.91–0.91) ^c^	0.87 (0.87–0.87) ^a,c^	0.92 (0.91–0.92) ^a,b^
Unstable	0.92 (0.92–0.92)	0.88 (0.88–0.88) ^a,c^	0.92 (0.92–0.93) ^b^
Deceased	0.94 (0.94–0.95) ^b,c^	0.95 (0.94–0.95) ^a,c^	0.99 (0.96–1.00) ^a,b^
Transition			
Unstable-Stable	0.77 (0.76–0.77) ^b^	0.70 (0.70–0.70) ^a^	0.73 (0.69–0.77)
Stable-Unstable	0.77 (0.76–0.77) ^c^	0.77 (0.77–0.77) ^c^	0.71 (0.67–0.75) ^a,b^
Life-sustaining therapy			
MV	0.80 (0.79–0.81) ^c^	0.81 (0.80–0.81) ^c^	0.73 (0.69–0.77) ^a,b^
VP	0.81 (0.80–0.82) ^b^	0.76 (0.76–0.77) ^a,c^	0.83 (0.78–0.87) ^b^
CRRT	0.94 (0.93–0.96) ^b^	0.86 (0.85–0.87) ^a^	0.93 (0.79–0.98)

Abbreviations: CRRT: Continuous Renal Replacement Therapy; MV: Mechanical Ventilation; VP: Vasopressors. Performance is the median AUROC across a 100-iteration bootstrap with 95% Confidence Intervals in parenthesis.

P-values are based on pairwise Wilcoxon rank sum tests.

a p-value < 0.05 compared to the development cohort.

b p-value < 0.05 compared to external cohort.

c p-value < 0.05 compared to prospective cohort.

**Table 3.** Mortality prediction performance of APRICOT-M compared to acuity baseline for episode and step levels.

Cohort	Model	Prevalence (episode)	AUROC (episode)	AUPRC (episode)	Prevalence (step)	AUROC (step)	AUPRC (step)

Development	SOFA	0.052	0.88 (0.87–0.89)	0.36 (0.33–0.38)	0.003	0.83 (0.82–0.84)	0.02 (0.02–0.02)
	APRICOT-M		**0.93 (0.93–0.94)** *	**0.55 (0.52–0.58)** *		**0.94 (0.94–0.95)** *	**0.22 (0.20–0.24)** *

External	SOFA	0.058	0.85 (0.85–0.85)	0.33 (0.32–0.35)	0.003	0.78 (0.78–0.79)	0.02 (0.01–0.02)
	APRICOT-M		**0.93 (0.93–0.94)** *	**0.59 (0.58–0.61)** *		**0.95 (0.94–0.95)** *	**0.23 (0.22–0.24)** *

Prospective	SOFA	0.027	0.92 (0.83–0.99)	0.28 (0.12–0.57)	0.001	0.80 (0.65–0.92)	0.00 (0.00–0.01)
	APRICOT-M		0.99 (0.97–1.00)	**0.78 (0.45–1.00)** *		**0.99 (0.97–1.00)** *	**0.48 (0.05–0.86)** *

Abbreviations: AUPRC: Area Under the Precision-Recall Curve; AUROC: Area Under the Receiving Operating Characteristic; SOFA: Sequential Organ Failure Assessment. Performance is the median AUROC and AUPRC across a 100-iteration bootstrap with 95% Confidence Intervals in parenthesis.

* p-value < 0.05 compared to SOFA.

**Table 4.** Instability prediction performance of APRICOT-M compared to acuity baseline for episode and step level.

Cohort	Model	Prevalence (episode)	AUROC (episode)	AUPRC (episode)	Prevalence (step)	AUROC (step)	AUPRC (step)

Development	SOFA	0.116	0.51 (0.50–0.52)	0.13 (0.13–0.14)	0.012	0.60 (0.60–0.61)	0.02 (0.02–0.02)
	SOFA (≥ 2 points)		0.49 (0.48–0.50)	0.28 (0.27–0.29)		0.54 (0.54–0.55)	**0.17 (0.16–0.17)**
	APRICOT-M		**0.73 (0.72–0.74)** **	**0.32 (0.31–0.34)** **		**0.74 (0.73–0.75)** **	0.10 (0.09–0.11)**

External	SOFA	0.131	0.46 (0.46–0.47)	0.13 (0.13–0.13)	0.013	0.55 (0.55–0.56)	0.02 (0.02–0.02)
	SOFA (≥ 2 points)		0.46 (0.46–0.46)	0.23 (0.22–0.23)		0.51 (0.51–0.51)	0.13 (0.12–0.13)
	APRICOT-M		**0.74 (0.74–0.75)** **	**0.47 (0.47–0.48)** **		**0.75 (0.74–0.75)** **	**0.24 (0.23–0.25)** **

Prospective	SOFA	0.242	0.61 (0.54–0.68)	0.39 (0.29–0.49)	0.011	0.68 (0.64–0.71)	0.02 (0.02–0.03)
	SOFA (≥ 2 points)		0.52 (0.46–0.58)	0.40 (0.33–0.48)		0.53 (0.50–0.57)	**0.12 (0.09–0.16)**
	APRICOT-M		0.61 (0.53–0.68)	0.41 (0.27–0.48)		**0.69 (0.64–0.74)** *	0.07 (0.04–0.10)**

Abbreviations: AUPRC: Area Under the Precision-Recall Curve; AUROC: Area Under the Receiving Operating Characteristic; SOFA: Sequential Organ Failure Assessment. Performance is the median AUROC and AUPRC across a 100-iteration bootstrap with 95% Confidence Intervals in parenthesis.

* p-value < 0.05 compared to one of SOFA or SOFA (≥ 2 points).

** p-value < 0.05 compared to SOFA and SOFA (≥ 2 points).

**Table 5.** APRICOT-M metrics at 33%-episode precision for episode and step-level predictions.

Outcome	Cohort	Earliest prediction (hours)	Number of early alerts	Sensitivity (episode)	Specificity (episode)	Sensitivity (step)	Specificity (step)	PPV (step)

Mortality	Development	20.00 (8.00–59.00)	2.00 (1.00–4.00)	0.76 (0.74–0.79)	0.92 (0.91–0.92)	0.57 (0.54–0.60)	0.99 (0.99–0.99)	0.10 (0.09–0.11)
	External	20.00 (8.00–68.00)	3.00 (1.00–5.00)	0.80 (0.79–0.82)	0.91 (0.91–0.91)	0.64 (0.63–0.65)	0.98 (0.98–0.98)	0.09 (0.09–0.09)
	Prospective	200.00 (68.00–444.00)	2.00 (1.00–2.50)	0.88 (0.63–1.00)	0.96 (0.94–0.97)	0.64 (0.00–0.95)	1.00 (1.00–1.00)	0.14 (0.00–0.31)

Instability	Development	8.00 (4.00–28.00)	1.00 (1.00–2.00)	0.36 (0.35–0.38)	0.90 (0.90–0.91)	0.21 (0.20–0.22)	0.98 (0.98–0.98)	0.12 (0.11–0.13)
	External	4.00 (4.00–16.00)	1.00 (1.00–2.00)	0.55 (0.54–0.56)	0.83 (0.83–0.83)	0.36 (0.36–0.37)	0.97 (0.97–0.97)	0.13 (0.13–0.13)
	Prospective	14.00 (8.00–41.00)	2.00 (1.00–2.00)	0.51 (0.41–0.61)	0.68 (0.62–0.74)	0.25 (0.16–0.31)	0.97 (0.97–0.97)	0.08 (0.05–0.11)

MV	Development	4.00 (4.00–20.00)	1.00 (1.00–2.00)	0.44 (0.42–0.46)	0.92 (0.91–0.92)	0.29 (0.28–0.31)	0.99 (0.99–0.99)	0.14 (0.14–0.15)
	External	4.00 (4.00–12.00)	1.00 (1.00–1.00)	0.60 (0.59–0.61)	0.85 (0.85–0.86)	0.46 (0.45–0.47)	0.97 (0.97–0.97)	0.14 (0.14–0.14)
	Prospective	18.00 (8.00–56.00)	2.00 (1.00–2.25)	0.63 (0.54–0.77)	0.64 (0.57–0.69)	0.35 (0.27–0.44)	0.96 (0.96–0.96)	0.08 (0.06–0.11)

VP	Development	4.00 (4.00–20.00)	1.00 (1.00–1.00)	0.44 (0.42–0.45)	0.90 (0.90–0.90)	0.28 (0.27–0.30)	0.98 (0.98–0.99)	0.16 (0.16–0.17)
	External	4.00 (4.00–8.00)	1.00 (1.00–1.00)	0.51 (0.50–0.52)	0.85 (0.85–0.85)	0.34 (0.33–0.34)	0.98 (0.98–0.98)	0.18 (0.18–0.18)
	Prospective	4.00 (4.00–4.00)	1.00 (1.00–1.00)	0.27 (0.16–0.40)	0.90 (0.86–0.93)	0.17 (0.11–0.26)	1.00 (1.00–1.00)	0.21 (0.14–0.31)

Abbreviations: MV: Mechanical Ventilation; PPV: Positive Predictive Value; VP: Vasopressors. PPV, sensitivity, and specificity are the median across a 100-iteration bootstrap with 95% Confidence Intervals in parenthesis. The earliest prediction and number of alerts are shown as the median (interquartile range).

**Table 6.** APRICOT-M performance for 4-h, 24-h, and 48-h predictions in terms of AUROC for three study cohorts.

Outcomes	Time Window	Development	External	Prospective

Primary				
Discharge	4-h	0.85 (0.85–0.86) ^c^	**0.85 (0.85–0.85)** ^b,c^	**0.86 (0.83–0.89)** ^b,c^
	24-h	0.85 (0.84–0.85) ^c^	0.79 (0.79–0.80) ^a,c^	0.70 (0.65–0.75) ^a^
	48-h	0.82 (0.81–0.83) ^a,b^	0.74 (0.73–0.75) ^a,b^	0.67 (0.61–0.73) ^a^

Stable	4-h	**0.91 (0.91–0.91)** ^b,c^	0.87 (0.87–0.87)	**0.92 (0.91–0.92)** ^b,c^
	24-h	0.89 (0.88–0.89) ^a,c^	0.86 (0.86–0.86) ^a^	0.84 (0.82–0.86) ^a^
	48-h	0.85 (0.85–0.86) ^a,b^	0.82 (0.82–0.82) ^a,b^	0.83 (0.80–0.86) ^a^

Unstable	4-h	0.92 (0.92–0.92) ^c^	0.88 (0.88–0.88) ^b^	**0.92 (0.92–0.93)** ^b,c^
	24-h	0.91 (0.91–0.91) ^a,c^	0.86 (0.86–0.87) ^a,c^	0.88 (0.86–0.89) ^a,c^
	48-h	0.88 (0.88–0.89) ^a,b^	0.80 (0.80–0.80) ^a,b^	0.85 (0.83–0.88) ^a,b^

Deceased	4-h	**0.94 (0.94–0.95)** ^b,c^	**0.95 (0.94–0.95)** ^b,c^	**0.99 (0.97–1.00)** ^b,c^
	24-h	0.91 (0.90–0.92) ^a,c^	0.88 (0.87–0.88) ^a,c^	0.90 (0.78–0.95) ^a^
	48-h	0.87 (0.85–0.88) ^a,b^	0.80 (0.80–0.81) ^a,b^	0.79 (0.64–0.91) ^a^

Transition				
	
Unstable-Stable	4-h	0.77 (0.76–0.77) ^b,c^	0.70 (0.70–0.70) ^b,c^	0.74 (0.70–0.77) ^c^
	24-h	**0.84 (0.83–0.84)** ^a,c^	**0.77 (0.77–0.78)** ^a,c^	0.70 (0.66–0.74) ^c^
	48-h	0.81 (0.80–0.82) ^a,b^	0.72 (0.71–0.72) ^a,b^	0.59 (0.55–0.64) ^a,b^

Stable-Unstable	4-h	0.77 (0.76–0.77) ^b^	**0.77 (0.76–0.77)** ^b,c^	0.71 (0.66–0.76) ^b^
	24-h	0.75 (0.74–0.77) ^a^	0.73 (0.72–0.74) ^a^	0.61 (0.54–0.68) ^a^
	48-h	0.76 (0.74–0.77)	0.73 (0.72–0.73) ^a^	0.61 (0.52–0.70)

Life-sustaining therapy				
	
MV	4-h	**0.80 (0.80–0.81)** ^b,c^	**0.81 (0.80–0.81)** ^b,c^	0.72 (0.68–0.78) ^c^
	24-h	0.77 (0.75–0.78) ^a,c^	0.75 (0.74–0.75) ^a,c^	0.64 (0.58–0.71)
	48-h	0.74 (0.72–0.76) ^a,b^	0.71 (0.71–0.72) ^a,b^	0.60 (0.52–0.68) ^a^

VP	4-h	**0.81 (0.81–0.82)** ^b,c^	**0.76 (0.76–0.77)** ^b,c^	**0.84 (0.79–0.87)** ^b,c^
	24-h	0.72 (0.71–0.73) ^a,c^	0.63 (0.63–0.64) ^a,c^	0.69 (0.60–0.74) ^a^
	48-h	0.67 (0.66–0.69) ^a,b^	0.58 (0.58–0.59) ^a,b^	0.63 (0.52–0.76) ^a^

CRRT	4-h	**0.95 (0.94–0.95)** ^b,c^	0.86 (0.85–0.87) ^b^	0.92 (0.79–0.98)
	24-h	0.93 (0.92–0.95) ^a^	**0.88 (0.88–0.89)** ^a,c^	0.74 (0.56–0.90)
	48-h	0.91 (0.87–0.94) ^a^	0.86 (0.85–0.87) ^b^	0.78 (0.52–0.97)

Abbreviations: CRRT: Continuous Renal Replacement Therapy; MV: Mechanical Ventilation; VP: Vasopressors. Performance is the median AUROC across a 100-iteration bootstrap with 95% Confidence Intervals in parenthesis.

P-values are based on pairwise Wilcoxon rank sum tests.

a p-value < 0.05 compared to a 4-h window.

b p-value < 0.05 compared to a 24-h window.

c p-value < 0.05 compared to a 48-hour window.

## Data Availability

The data from MIMIC and eICU is publicly available and can be accessed through PhysioNet at the following links after obtaining approval: MIMIC-IV (https://physionet.org/content/mimiciv/2.2/) and eICU-CRD (https://physionet.org/content/eicu-crd/2.0/). The data from UFH is private and has not been approved for public use. The code for running all data processing, model training/validation, and analyses, is freely available at https://github.com/iheallab/apricotM. All analyses carried out in this study were performed using Python version 3.10.8. The Python package torch version 2.1.2, mamba-ssm 1.1.1, and scikit-learn 1.4.0 was used for developing the machine learning and deep learning models. Integrated gradients were implemented using the Python package Captum version 0.7.0. Visualization plots/graphs were obtained using Python packages matplotlib version 3.8.3 and seaborn version 0.13.2. Statistical analyses were performed using scipy version 1.12.0.
